# Leukocytosis in Surgical Infections1Read at the thirty-ninth annual meeting of the Medical Association of Central New York, at Syracuse, October 16, 1906.

**Published:** 1907-04

**Authors:** Charles O. Boswell

**Affiliations:** Rochester, N. Y.


### Leukocytosis in Surgical Infections.[1]

By CHARLES O. BOSWELL, M. D., Rochester, N. Y.

FOR many years the fact that in most cases of infections, the blood shows an increase in the number of the leukocytes has been known. The phagocytic theory of Metchnikoff well explains this phenomena, and the polynuclear neuthrophiles having the greatest phagocytic power were found to be responsible for this increase, the vast majority of the leukocytes being of this type. Yet, in not every infectious disease is there a leukocytosis, nor does it always increase proportionately to the extent of the infection.

Certain bacteria, such as the b. typhosus, do not seem to have the power to arouse in the blood stream an increase of the leukocytes. Again, certain factors favoring a virulent course of the infectious element may give rise to such an intense toxemia that the resisting power of the body is overcome and the blood may show only a slight leukocytosis. The theory of opsonins, lately given us by Wright, promises much that will further elucidate the problem of the infections.

It having been established as a well nigh universal rule that in the presence of a septic infection the blood shows an increase of the leukocytes, which increase is almost entirely composed of the polymorphonuclear cells, it was soon advocated as a diagnostic means to reckon the number of the leukocytes in the circulating blood and to repeat this procedure at varying intervals, believing that in this way some indication of the progress of the infection could be obtained.

Occasionally operative measures or autopsy showed that conclusions formed in this manner were not always correct. While generally the numbers of the leukocytes present in these acute surgical infections were in direct proportion to the extent of the infectious process and the power of the patient's resistance, occasionally no leukocytosis, or only a mild one, could be demonstrated; yet the most widespread septic conditions were revealed on opening the body, and this sometimes with only slight clinical symptoms. Or, again, where the patient clinically showed a most marked picture of sepsis, or a well walled off collection of pus would not give rise to leukocytosis! Such experiences seemed to cast a damper on the value of the number of leukocytes as an aid to diagnosis in these conditions.

The counting of stained blood smears had shown the disproportionate increase in the polymorphonuclear elements; still there was no absolute indication of the extent of the infection

1. Read at the thirty-ninth annual meeting of the Medical Association of Central New York, at Syracuse, October 16, 1906.

even here. There has not been found any definite proportion of polymorphonuclear cells to serve as the point at which suppuration may be said definitely to be present.

It was found that when suppuration or gangrene was definitely established that the proportion of these cells was often over 90% of all leukocytes, and with diminishing infection a steady decrease could be determined; also that a maintained or steadily progressing increase of the leukocytes, associated with a proportionate increase of the polymorphonuclear cells, was of good omen. With the recovery of the patient, the leukocytes would gradually lessen.

Gibson, writing for the April number of the Annals of Surgery, in an elaborate article claims great weight for the proportionate increase of the polymorphonuclear cells, and rightly claims a greater weight for this proportionate increase than for the increase in the total number, this phenomena meaning to him either a severe lesion, that is, an active infection or absorption, or a toxemia, or both. He shows a chart having a base line reading at 10,000, the high normal limit for leukocytes, and other line indicating 75% of polymorphonuclear cells, again the high normal value. By plotting this chart much as a fever chart is plotted, the parallelism of the factors is readily shown. This gives a guide to prognosis as well as furnishes an indication for or against operation.

While considerable time and attention has been given to the percentage of the polymorphonuclear cells and to the total number of the leukocytes, but little attention has been given to the other varieties of the white cells. The fall in proportion of the mononuclear cells, of course was noticed; but so far, no definite conclusions have been based on this factor.

Holmes, of Denver, read a paper before the section on Pathology and Physiology of the American Medical Association in June, 1904, in which he endeavored to show that the small mononuclear cells being nonphagocytic, served as an index of the patient's resistance and of the strength of the infection. This view is very different from what would naturally follow, the idea of phagocytosis being concerned chiefly with the polynuclear cells.

Another white blood cell—the eosinophile—has only recently been considered in connection with infections. Simon, of Baltimore, in the last edition of his textbook, published in 1904, made this statement regarding septic infections: "The neutrophiles are relatively increased and the eosinophiles very much diminished, or absent altogether, except in osteomyelitis, which is an exception as regards the eosins."

About 18 months ago I had the privilege of working in Simon's laboratory, where, at that time, he was further investigating the phenomena. He showed stained smears of blood from various septic conditions, all of which showed this change. It was not, however, until this year that he published the results of his experiments. The article appeared in the first number of the International Clinics for 1906 and is entitled, "A study of the Occurrence of Hypœosinophilia in Septic Infections." In this article he calls attention to the fact that in previous communications on the subject of blood in infectious diseases, while noting the relative increase of the polymorphonuclear cells, nothing has been said of the eosinophiles, thus overlooking the most important point,—namely, the increase of the polymorphonuclear cells associated with a decrease, or even a complete absence, of the eosinophiles. This phenomena he calls "the septic factor." He gives a list of 41 cases, all of septic nature, half of them being cases of appendicitis. In no case was the percentage of the polymorphonuclears below 76, while the percentage of the eosinophiles was never above .4. The experimental work was done on guinea pigs, and he found the same law to hold good. He concludes that the active agent producing this change is an intracellular ferment.

The percentage of polymorphonuclear cells, as given by him, is from 60-70, with 74 as the high normal limit; of the eosinophiles, 2-4. A further practical point noted by him, regarding the eosinophiles, is that as soon as convalescence is reestablished, they reappear in the blood and gradually assume a percentage which may be higher than normal, the so-called "epicritic eosinophilia." By this change a guide to beginning reaction is furnished.

For the past 15 months I have made numerous blood counts, having this phenomena in mind, and have yet to see it fail. The patients were, all but one, at the Rochester City Hospital,—both ward and private. I shall briefly report a few of these cases:

*Case I.* Miss G. L., private patient of Dr. Whitbeck, admitted to the hospital February 16, 1906; ill six days; diagnosis, appendicitis. Total leukocytosis 16,000. A differential count gave,

| | | |
|---|---|---|
| Small mononuclears | 50 | 9.3% |
| Large mononuclears | 25 | 4.7% |
| Polymorphonuclears | 425 | 85.3% |
| Mast cells | 1 | .1% |
| Eosinophiles | 2 | .3% |

Operation showed beginning gangrene of appendix; some portions of the ileum were ecchymotic and covered with lymph; no free pus. The wound was closed without drainage. Here was a fairly severe condition, with but a slight leukocytosis, but the altered interrelation between the polymorphonuclears and the eosinophiles, referred to as the septic factor, is readily seen. On February 19, total leukocytosis 14000, polys. 78.1%, eosins.

.1%. This is a decrease in both these values. The patient was not doing very well, being still septic with intermittent temperature and rapid pulse. On February 26, the patient was much better; temperature normal. Total leukocytosis 12,600, polys. 83%, eosins. increasing now 1%.

*Case 2.* Mrs. G. S., admitted to the hospital January 29, 1906. She had been ill 8 days; pain at first diffuse, later localised in right iliac region. Pain had suddenly ceased four days before admission, but fever and constitutional symptoms increased. Temperature on admission 101 4-5 degrees. Total leukocytosis 27,000, polys. 85%, eosins. 0. Operation by Dr. E. W. Mulligan. A gangrenous appendix was found, lying in a pool of pus and beginning general peritonitis. February 2, total leukocytosis 26,000, polys. 87%, eosins. 0. The paralysis of the the bowels, which had been present, was relieved today. February 5, no total leukocyte count, but the stained smear showed polys. 90%, eosins. 0. The patient died that night.

*Case 3.* Mrs. E. K. Seen by myself in consultation. This patient had undoubtedly produced an incomplete abortion on herself. The second day after flooding began her physician did a manual extraction of a dead fetus about four months old. Two days later signs of a beginning peritonitis appeared with great distention of the abdomen and complete obstruction of the bowels. I saw her on February 2, 1906, when fecal vomiting had existed for several hours. I did not make a total count, but the percentage of polys. in the stained specimen was 86, with no eosins. The urine gave a most marked reaction for indican. The patient was removed to the Homeopathic Hospital, where Dr. F. W. Zimmer performed an enterostomy. There was marked general peritonitis. Death resulted in a few hours. No further blood examinations were made.

*Case 4.* Mrs. E. S. Admitted to hospital February 17, 1906; service of Dr. Whitbeck. This patient had miscarried about 6 months ago. Soon afterward says she was ill with typhoid fever in a hospital (location not given), for about 16 weeks. As a result of this illness her joints, especially the left knee and the vertebra articulations, were left stiff, and for this reason she had employed massage freely up to a few days ago. On the day before admission she suffered from severe pain in the abdomen, gradually becoming localised in the right iliac region. Vaginal examination showed some tenderness in the region of the right ovary and complaint of pain throughout bowels. A blood count made by Dr. Witherspoon showed 20,000 leukocytes, polys. 76%, no eosins. Operation revealed a large, firmly adherent appendix, containing pus. There was also some pus about the appendix, so drainage was established. On February 18, total leukocytosis 16,400, polys. 89.7%, eosins. 0. On February 19, total leukocytosis 12,400, polys. 76.5%, eosins. .5%. Free drainage was now well established. On February 26, total leukocytosis 11,600, polys. 80.6%, eosins. 1%, and 2

normoblasts had somehow strayed in the field. Improvement was now so marked that the drainage tubes were removed.

*Case 5.* E. H. Admitted February 23, 1906; service of Dr. Hastings. Present illness began on the day previous with chill and pain in the abdomen. On admission the abdomen was distended, with tenderness in right iliac region, but no rigidity. Total leukocytosis 18,600, polys. 90.5%, eosins. 0. The appendix was found to be gangrenous with some free pus about it.

*Case 6.* A private patient of Dr. E. O. Jones, was admitted March 8, 1906. Diagnosis appendicitis. Total leukocytosis 14,200, polys. 90.4%, eosins. 0. Operation revealed a sloughed gangrenous appendix, with general purulent peritonitis. The patient died soon afterward. The operation was performed while I was making the differential count. I am told that I had stated —after making the total count—that I believed the low leukocyte count excluded pus. I do not remember having made any such statement, but if I did, it will serve as a warning not to be too free with opinions when I have only partially made a count. Certainly the septic factor is well established here and I would not have excluded sepsis after the differential count.

*Case 7.* Agnes S. Seen by me at the medical clinic of the O. P. D., July 17, 1906. She walked into the clinic with her mother, giving a history of illness of barely 24 hours. She had slight abdominal pain, with vomiting. The pain was becoming localised in the right iliac region; no tumor; slight rigidity of right rectus muscle on firm palpitation. Transferred to children's ward for observation. Temperature 99 3-5 degrees, pulse 130. This combination of rapid pulse rate out of proportion to the extent of fever. when there are suggestive symptoms, seems to me to be a most important point in the symptomology of appendicitis. Total leukocytosis 13,000, polys. 87%, eosins. 0. The pain in the region of the appendix becoming more acute, Dr. H. T. Williams operated at midnight, about 12 hours after the child was admitted. There was beginning gangrene, the appendix was swollen and surrounded by a plastic exudation. This case was most interesting. The apparent trifling illness of the child when she came to the clinic and the mildness of the pain made the diagnosis of appendicitis seem rather tentative, yet an operation only 12 hours later revealed an extreme extent of disease. The very low leukocytosis here would have misled if only the total count had been considered. No case illustrates the value of the septic factor better.

*Case 8.* Mrs. M. F. Admitted to the service of Dr. Whitbeck, June 6, 1906. Diagnosis puerperal sepsis. Patient was delivered May 30, and had a chill the following day. Leukocytes on admission 14,000, polys. 80%, eosins. 0. On June 8, total leukocytosis 36,000, polys. 85.4%, eosins. .3%. On June 10, the total leukocytosis had fallen to 10,000, polys. 91.8%, eosins. 0. There was extreme anemia, the red cells being very pale and exhibiting great irregularity in size and shape. Attempt at blood culture proved negative, I being scarcely able to obtain any blood from the veins at the elbow. The patient was rapidly sinking and died June 15.

*Case 9.* D. E. Ward patient, service of Dr. Rose. Was operated Sept. 21, 1905, for suppurative appendicitis. No blood count was made previous to operation. Free pus was found and drainage tubes inserted. The following day the total leukocytosis was 11,500, polys. 82.6%, eosins. 0. On Sept. 24, total leukocytosis 11,700, polys. 82.3%, eosins. 2.6%. Free drainage going on, temperature normal. Sept. 27, total leukocytosis 12,-500, polys. 75.7%, eosins. 6.6%, which is above the normal average. This hypereosinophilia is a common phenomena of recovery from infectious states and may, at times, reach as high as 10-12%. The discovery of eosinophiles is a matter of considerable encouragement for prognosis, as Simon has pointed out. On October 3, the total leukocytosis was 6,200, polys. 60%, eosins. 7.2%. The pulse, temperature and respirations had now been normal for 9 days. On October 11, no total count was made, polys. 70%, eosins. 6%. Patient discharged cured. This case illustrates beautifully the occurrence of an increase of eosinophiles as convalescence progresses.

In conclusion I wish to call attention to these findings:

1. The total leukocytosis, especially if but one count be made, is of little value and may mislead entirely in forming an opinion as to the extent of the infection.

2. There is no absolute number of leukocytes which answers definitely the question, "Is suppuration or gangrene already at hand"; nor any fixed percentage of polynuclear cells.

3. In these few cases gangrenous changes were often accompanied by a lower total leukocytosis than when pus outside the appendix was found.

5. It is not the percentage of the polymorphonuclear cells alone that is an index to the recuperative powers of the body, but the increase or reappearance of the eosinophiles that form the basis for prognosis.

6. Beginning improvement is always announced by a reappearance in the blood stream of the eosinophiles, which may exceed their normal values. Unfavorable termination is to be expected when these cells remain continuously absent and the percentage of the polymorphonuclear cells may slowly fall in such an event.

41 Gibbs Street.

---

R. Cruchet and Lepage (*Annales de médecine et de chirurgie infantiles,* February, 1905) point out that as infants never expectorate but always swallow sputa, it is of great importance to examine their stools for the bacillus of Koch in cases where tuberculosis is suspected.—*St. Louis Medical Review.*

